# Electrical impedance tomography-based temporal signals correlate with quantitative computed tomography-based spatial variables in asthma subjects: a pilot study

**DOI:** 10.3389/fphys.2025.1660948

**Published:** 2025-08-22

**Authors:** Jinyoung Jeong, Yuna Kim, Seung Eun Lee, Hye Ju Yeo, Sungchul Huh, Sanghun Choi

**Affiliations:** ^1^ School of Mechanical Engineering and IEDT, Kyungpook National University, Daegu, Republic of Korea; ^2^ Research Institute for Convergence of Biomedical Science and Technology, Pusan National University Yangsan Hospital, Yangsan, Republic of Korea; ^3^ Department of Internal Medicine, Pusan National University Yangsan Hospital, Yangsan, Republic of Korea; ^4^ Department of Rehabilitation Medicine, Pusan National University Yangsan Hospital, Yangsan, Republic of Korea

**Keywords:** computed tomography, electrical impedance tomography, asthma, fast fourier transform, pulmonary function tests

## Abstract

**Introduction:**

Quantitative computed tomography (qCT) provides detailed spatial assessments of lung structure and function, while electrical impedance tomography (EIT) offers high temporal resolution for analyzing breathing patterns but lacks structural detail. This study investigates the correlation between qCT-based spatial variables and EIT-based temporal signals to elucidate the physiological relationships between these two modalities.

**Methods:**

Six participants with asthma underwent pulmonary function tests (PFTs) before and after bronchodilator inhalation. CT scans were obtained in full inhalation and normal exhalation, providing airway hydraulic luminal diameter (*D*_h_), airway wall thickness, and percent emphysema, and percent functional small airway disease (fSAD%). On the same day, EIT imaging was performed during tidal breathing, measuring impedance in three different anatomical regions on the chest wall (upper, middle, and lower). The EIT-based impedance was transformed using fast Fourier transform (FFT) to separate perfusion and respiration components including high and low frequencies.

**Results:**

High-frequency EIT values in the upper lungs were associated with a decline of lung function in pre-bronchodilator. Skewness values, measured by an imbalance between exhalation and inhalation, was elevated in the upper lungs and correlated with impaired lung function. Besides, *D*_h_ decreased with a higher expiration-to-inspiration (E:I) ratio in the upper lungs and middle lungs. Functionally, higher high frequency values and lower low frequency values in the upper lungs correlated with greater emphysema and functional small airway disease, accompanied by reduced ventilation deformation metrics. Similarly, increased hysteresis variables (e.g., E:I, skewness) in the upper and middle lungs were associated with a further decrease in ventilation deformation metrics.

**Conclusion:**

EIT temporal signals demonstrated significant associations with spatial metrics from CT images, as well as PFTs metrics. A frequency analysis of EIT may enhance diagnostic approaches and improve understanding of respiratory mechanics in subjects with asthma.

## Introduction

Asthma is characterized by episodic symptoms of airway narrowing that make breathing difficult. These symptoms in asthma appear repeatedly and episodically, caused by a combination of genetic and environmental factors (Barnes and Drazen, 2002). The inflammation of the airways causes the airway mucosa to swell and the airway muscles to contract, leading to obstruction and impaired airflow (Tillie‐Leblond et al., 2009). Typical symptoms in obstructive airways include dyspnea, coughing, and wheezing (Vestbo et al., 2013). Under normal conditions, human respiration involves relatively short inhalations and comparatively longer exhalations (Barnes et al., 1998; McCracke et al., 2017). Temporal breathing patterns can reflect alterations in segmental large airways, parenchymal small airways, and even perfusion (Deibele et al., 2008; Zadehkoochak et al., 1992; Noordegraaf et al., 1998). Due to its high temporal resolution, electrical impedance tomography (EIT) offers unique insights into such pathophysiology (Brovman et al., 2018; Cao et al., 2020; Mansouri et al., 2021).

Meanwhile, with the advantage of high spatial resolution, quantitative computed tomography (qCT) images can extract structural imaging characteristics such as the hydraulic luminal diameter (*D*_h_) and airway wall thickness (WT) of the airways (Hoffman et al., 2006; Choi et al., 2015). In addition, parenchymal functional characteristics such as Jacobian and functional small airway disease (fSAD) can be extracted by applying image registration technique with exhalation and inhalation images (Choi et al., 2017; Choi et al., 2013; Jeong et al., 2024; Pyo et al., 2023; Choi et al., 2014). While CT images have proven to be useful in characterizing disease alterations sensitively with its own high spatial resolution, it is still arguable if CT images acquired in the static states provide dynamic features during actual breathing over time (Wongviriyawong et al., 2013). In contrast, the EIT is suitable for analyzing characteristics that subtly change over time, such as breathing patterns in asthma patients. However, it can limit detailed imaging-based pathophysiology due to its poor image resolution.

In a previous EIT study, Frerichs et al. (Frerichs et al., 2009) employed band-pass filtering techniques and linear regression fitting to delineate functional regions of interest (ROI) within the left and right lungs, in mechanically ventilated subjects. Fagerberg et al. (Fagerberg et al., 2009a; Fagerberg et al., 2009b) explored the EIT method to measure perfusion impedance during apnea, but it could not fully address the complex interplay between ventilation and perfusion. In response to these limitations, Caroline A. Grant et al. (Grant et al., 2011) proposed a step-by-step approach based on previous filtering techniques. This method extends its applicability to a spontaneous breathing cohort and enables analysis of ventilation-perfusion relationships. Consequently, the EIT could measure the amount of ventilation by estimating the amount of change in the patient’s impedance, and visualize the movement of the lungs by expressing this as an image. In addition, perfusion signals can be detected by measuring the vibration of minute impedance signals.

Thus, EIT is recognized as a valuable technology for providing complementary insights into patients’ lung function alongside conventional pulmonary function tests (PFTs), as it offers regional information and is less sensitive to patients’ compliance. Recently, lung EIT research has been actively conducted to improve the resolution of EIT images or to generate 3D images by utilizing image reconstruction algorithms (Gao et al., 2024). An EIT study has demonstrated a local lung function decline in asthma patients (Frerichs et al., 2016), compared with CT at the voxel level qualitatively. However, it has still yet to confirm if the structural and functional variables obtained from static CTs sufficiently reflect the dynamic signals obtained from EIT. Accordingly, we hypothesized that various structural and functional variables extracted from static CT images reflect dynamic characteristics measured from EIT temporal signals.

In this background, this study focused on the signal of impedance change over time rather than the spatial resolution of the EIT image, investigating the correlation between EIT images and CT scans. To achieve these research objectives, the data obtained through CT and EIT were compared and analyzed to confirm the relationship with lung function. This provides an insight into how CT and EIT can play complementary roles, and also how to integrate the two techniques to achieve better lung function evaluation. These findings are expected to present new prospects for the diagnosis and treatment of lung disease.

## Materials and methods

### Demography and pulmonary function tests of patients recruited

This study was designed as a hypothesis-generating pilot study, with the primary goal of evaluating the feasibility of integrating two complex advanced imaging techniques, Electrical Impedance Tomography (EIT) and quantitative Computed Tomography (qCT), and generating preliminary data for the design of future large-scale validation studies. The number of participants that could be recruited was limited by the realistic constraints of a 1-year institutional research project. Institutional review board of Pusan National University Yangsan Hospital has approved the current study (20-2021-007). EIT scanning was conducted at Pusan National University Yangsan Hospital, South Korea, with six asthma patients aged 49–68 years. Among the six participants, five met the criteria for severe asthma according to the 2023 GINA guidelines, and none had any other respiratory diseases. All participants used inhaled corticosteroids (ICS), with one additionally receiving oral corticosteroids (OCS). Three participants were also on concomitant bronchodilator. Detailed clinical information for each participant is presented in Table 1. Pulmonary function tests (PFTs) were performed to assess parameters, including forced expiratory volume in one second (FEV1), forced vital capacity (FVC), the ratio of FEV1 to FVC (FEV1/FVC), and forced expiratory flow between 25% and 75% of vital capacity (FEF25%-75%). Consistent with the American Thoracic Society guidelines for reproducibility, PFT values that showed significant variability or did not meet quality control criteria were excluded as outliers. The highest valid measurement among repeated tests was retained for analysis. All PFT assessments were conducted with patients seated upright to ensure standardized conditions. Each test was performed three consecutive times, allowing sufficient rest periods between trials, and the maximum value was selected for final analysis. PFT was measured in milliliters (mL) using the Vmax 22 (SensorMedics, USA). The PFTs were administered and supervised by a board-certified respiratory therapist to maintain measurement consistency and accuracy (Graham et al., 2019; Crapo et al., 1995). These parameters were measured both before and after bronchodilator administration for comparison with electrical impedance tomography (EIT) measures.

**TABLE 1:** Medical records of each subject.

Characteristic	Subject 1	Subject 2	Subject 3	Subject 4	Subject 5	Subject 6
**Sex**	**Male**					
Age	64	49	62	68	61	61
Height	168	175	182	163	165	164
BMI	23	35	27	25	24	23
Severity	O	O	O	O	X	O
Comorbidity	X	X	X	X	X	X
Medication use						
LAMA	Glycopyrronium	Glycopyrronium	Glycopyrronium	Umeclidinium	-	Umeclidinium
LABA	Indacaterol	Indacaterol	Formoterol	Vilanterol	Formoterol	Vilanterol
SABA	Salbutamol	-	Salbutamol	-	-	-
ICS	Budesonide	Budesonide	Beclometasone	Fluticasone	Budesonide	Fluticasone
OCS	Methylprednisolone	-	-	-	-	-
Blood/serum						
Total WBC, N/µl	10.4	8.51	7.53	5.89	11.39	13.85
% Eosinophils	0.4	2.4	1.5	2	0	0.1
% Neutrophils	73.6	60.3	61.3	53.7	91.7	71.3
% Lymphocyte	15.2	28.6	29.9	37.4	5.5	23.5
Additional biomarkers						
Total IgE, IU/mL	426	>2000	1381	34.81	165	653
FeNO, ppb	103	25	19	N/A	16	30
Exacerbation History						
ICU Hospitalized in the past year	0	0	0	0	0	0

### Acquisitions of EIT and CT images

**FIGURE 1:**
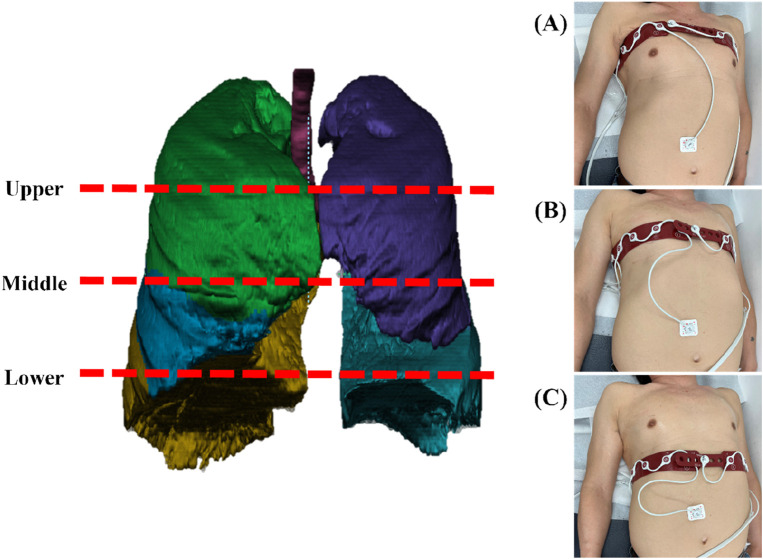
An illustration of three different anatomical regions on the chest wall where EIT scanned: **(A)** upper lung as measured on a patient, **(B)** middle lung as measured on a patient, and **(C)** lower lung as measured on a patient.

Electrical Impedance Tomography (EIT) measurements were performed using a PulmoVista® 500 device (Dräger Medical, Lübeck, Germany). The system uses a flexible silicone belt containing 16 integrated stainless steel electrodes, which was placed around the patient’s thorax. An additional reference electrode was attached to the central abdominal region to ensure a stable potential baseline. Data were acquired using a 4-terminal (tetrapolar) measurement protocol, which minimizes the effects of electrode-skin contact impedance. A low-amplitude, high-frequency alternating current (AC) was applied using a single frequency standard for ventilation monitoring. The device employs an adjacent injection pattern, where current is passed between neighboring electrodes, and the active pair is rotated sequentially around the thorax to generate a full data frame. Data were acquired continuously at a frame rate of 10 frames per second (fps) to ensure high temporal resolution for analyzing respiratory dynamics. To ensure data quality, electrode-skin contact impedance was continuously monitored by the device’s automated quality control system. The electrodes were positioned on the chest wall at three distinct anatomical regions corresponding to the upper, middle, and lower lungs (Figure 1). This selection reflects the anatomical division of the right lung into upper, middle, and lower lobes, and the left lung into upper and lower lobes, thereby capturing the functional characteristics of each pulmonary lobe. The rationale for this three-plane setup was not to achieve the anatomical precision of CT but to capture the well-known vertical heterogeneity of lung function. This approach provides a simplified model for assessing these vertical functional gradients. The upper thorax reflects thoracic breathing, characteristic of spontaneous respiration, the middle thorax encompasses the influence of major airways and vascular structures, and the lower thorax is strongly affected by diaphragmatic motion and gravity-dependent ventilation-perfusion effects.

EIT data were recorded during tidal breathing for over 60 s while patients were in the supine position. The measurement was initiated in a relaxed state to ensure stable breathing patterns. A stable 60-s respiration period from the patient’s first cycle of breathing was selected for analysis. Tidal breathing predominantly influences the upper lung regions, while gravitational effects contribute to ventilation heterogeneity across vertical positions. The upper thorax reflects thoracic breathing, characteristic of spontaneous respiration, the middle thorax encompasses the influence of major airways and vascular structures, and the lower thorax is strongly affected by diaphragmatic motion and gravity-dependent ventilation-perfusion effects (Bastir et al., 2017). This stratified electrode placement provides adequate spatial resolution without prolonging measurement time or causing patient discomfort. Data obtained from the three vertical positions were sufficient to evaluate the functional characteristics of each pulmonary lobe, allowing for the assessment of anatomical and functional vertical inhomogeneity in lung function. This comprehensive approach demonstrates that the selection of three vertical positions is supported by both scientific rationale and practical considerations.

An alternating current was injected through a selected pair of electrodes, and surface potentials were measured across the remaining electrode pairs. Bioelectrical impedance was calculated using Ohm’s law. The electrode pairs sequentially rotated around the thorax, yielding 16 voltage profiles. Each profile consisted of 13 voltage measurements, resulting in 208 data points used for reconstructing cross-sectional images. EIT image reconstruction facilitated by dedicated software (MATLAB® 914.0, The MathWorks Inc., Natick, MA, USA, and EIDORS 3.11, Open Source). Baseline measurements were automatically established using a reference measurement system. To ensure the integrity and reliability of the measured physiological signals, electrode-skin contact impedance was continuously monitored by the device’s automated quality control system. Monitoring contact impedance is a critical technical quality control measure that is a prerequisite for obtaining reliable physiological data; it is not a physiological measurement itself. EIT operates by injecting a small, precise alternating current and measuring the resulting millivolt-level surface potentials. If the contact impedance between the electrodes and the skin is too high or unstable, it can distort the injected current field and introduce significant noise into the sensitive voltage measurements, rendering the subsequent data unreliable. Therefore, this procedure verifies a stable and high-quality connection between the electrodes and the patient, which is essential for minimizing artifacts and measurement inaccuracies in the acquired voltage data. A multi-stage process was implemented to minimize measurement inaccuracies from artifacts. First, at the source, the device’s automated quality control system continuously monitored electrode-skin contact impedance to ensure high signal quality. Second, to exclude gross motion artifacts, the analysis was performed on a 60-s segment of data that was selected from a longer recording after visual inspection for a stable tidal breathing pattern, free from coughs or significant body movements. Finally, as detailed in the signal processing section, a Fast Fourier Transform (FFT)-based filtering technique was employed to separate the low-frequency impedance changes associated with ventilation from the higher-frequency signals associated with cardiac perfusion.

An image reconstruction was performed using a Finite Element Method (FEM)-based linearized Newton-Raphson algorithm. This algorithm first reconstructs impedance changes onto an FEM mesh composed of non-uniform triangular elements that model a thoracic cross-section. For visualization and subsequent analysis, the impedance values from this irregular mesh were then interpolated onto a regular 32 × 32 resolution grid to create the final image matrix. Reconstruction artifacts—particularly noise and instability—can arise near the image boundaries, especially in regions adjacent to the electrodes. To address these artifacts, we applied a selective boundary filtering technique as a post-reconstruction step. This spatial filter specifically targets peripheral elements in the reconstructed image, smoothing them to reduce boundary-related artifacts and enhance the signal-to-noise ratio in the central lung regions, which are of primary clinical interest. Subsequently, linear interpolation was performed to produce a smooth and continuous image suitable for visual interpretation.

On the same day these measurements were made, PFTs and high-resolution CT scans were recorded simultaneously. The CT scans were acquired in the supine position using a consistent imaging protocol on two SIEMENS Definition scanners (SOMATOM Definition Flash, Siemens Healthineers, Germany; SOMATOM Definition AS+, Siemens Healthineers, Germany) to mitigate inter-scanner variability. The scans were conducted both at total lung capacity (TLC) and functional residual capacity (FRC). High-resolution CT (HRCT) helical protocol was employed throughout with a standardized slice thickness of 0.6 mm. Structural variables derived from these CT scans underwent normalization to enhance comparability across subjects. Detailed information on the imaging protocol is provided in Table 2.

**TABLE 2:** Scanner and scanning protocol for CT imaging.

Parameter	Specification
Institution	PNUYH
Scanner model	SIEMENS Definition Flash or SIEMENS AS+
Scan type	Helical
Rotation time (s)	1
Detector configuration	1085.6
Pitch	1.5
Peak kilovoltage (kVp)	120
mAs	100
Dose modulation	None
Reconstruction algorithm	Mediastinum and Lung
Thickness (mm)	0.6
Iterative reconstruction	None

### CT-based structural and functional variables

**FIGURE 2:**
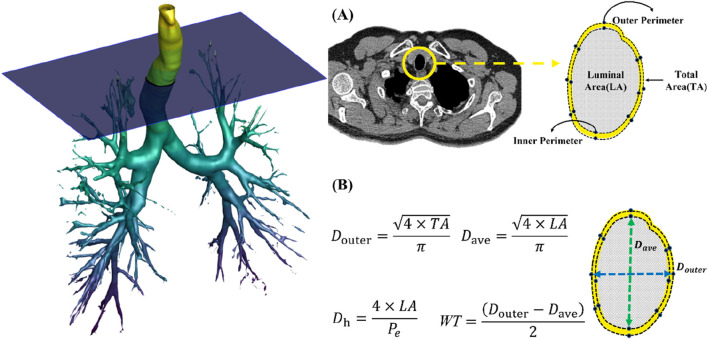
**(A)** Extraction of luminal area, inner perimeter and outer perimeter. **(B)** A schematic of obtaining hydraulic diameter (*D*_h_) and wall thickness (WT).

For the CT image analysis of both TLC and FRC scans, a commercial segmentation software AVIEW (Corline Soft, Co., Ltd., Seoul, Republic of Korea) was used, followed by image post-processing to extract the desired morphological features of the bronchial structure, including hydraulic luminal diameter (*D*_h_) and airway wall thickness (WT) (Choi et al., 2015). The bronchial tree was automatically segmented and manually labeled according to anatomical airway structures, allowing for accurate measurement of these variables, calculated using Equations 1a, 1b, respectively:

$$D_{h}=\frac{4\times LA}{P_{e}}$$ (1a)

$$WT=\left(D_{outer}-D_{ave}\right)\;/\;2$$ (1b)

where *LA*, *P*_e_, *D*_outer_, and *D*_ave_ are luminal cross-sectional area, perimeter of the cross-section, outer diameter, and luminal diameter (Figure 2). The *D*_h_ and WT were respectively normalized based on predicted values from healthy Korean individuals (Chae et al., 2021), using Equations 2a, 2b:

$$D_{trachea}=12.79-0.13\log\left(age\right)-5.82\log\left(height\right)\times sex+3.01\log\left(age\right)\times \log\left(height\right)$$ (2a)

$$WT_{trachea}=\log\left[9.11-1.02\log\left(age\right)-0.98\;{height}^{2}\;\times sex+1.01\,{height}^{2}\times \log\left(age\right)\right]$$ (2b)

**FIGURE 3:**
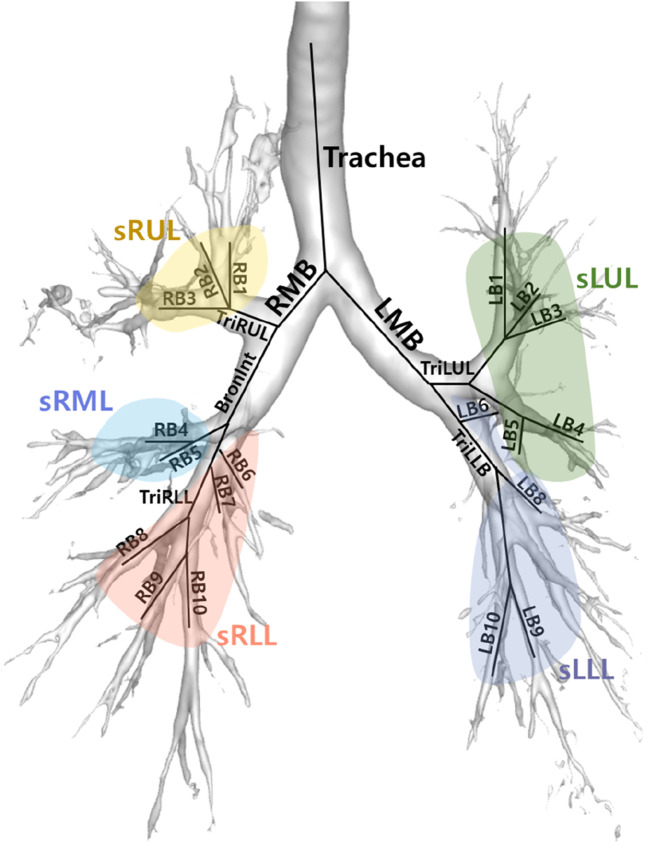
Location of 26 segmental airways and their respective five subgroups.

The *D*_h_ and WT were measured at the trachea, left and right main bronchi, right intermediate bronchus (Bronint), and the four bronchi (TriRUL, TriLUL, TriRLL, and TriLLB) leading to the lobes. Furthermore, *D*_h_ and WT values were acquired from the five subgroups within the lobes. Figure 3 depicts the labeling of 26 segmental airways and the categorization into five subgroups of lobes for the extraction of structural variables.

**FIGURE 4:**
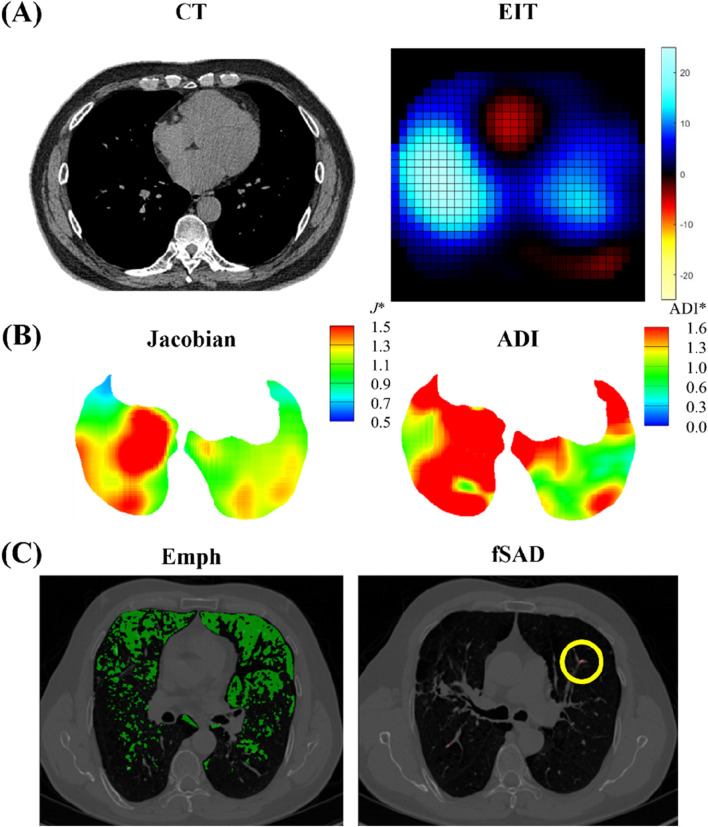
Illustrations of **(A)** CT and EIT images; **(B)** image registration-derived metrics of Jacobian and ADI; **(C)** emphysema (Emph) and functional small airway disease (fSAD) based on Hounsfield Unit values.

Figure 4 illustrates original images acquired from static CT and EIT. From an image registration with two static CT images, we further acquired parenchymal functional variables, including the proportion of abnormal regions characterized by CT Hounsfield units (HU), focusing on fSAD (under −856 HU) and Emphysema (under −950 HU) (Schroeder et al., 2013). The image registration also provides the determinant of Jacobian (Jacobian) and the anisotropic deformation index (ADI) (Choi et al., 2017; Kim et al., 2022). The Jacobian determinant, a scalar value calculated for each voxel, quantifies the local volume change of lung parenchyma between the FRC and TLC states. It is derived from the deformation field that maps the FRC image to the TLC image and serves as a direct measure of regional lung expansion (Equation 3a). The anisotropic deformation index (ADI) was also calculated (Equation 3b).

$$\text{Jacobian}=\lambda_{1}\lambda_{2}\lambda_{3}$$ (3a)

$$\text{ADI}=\sqrt{{\left(\frac{\lambda_{1}-\lambda_{2}}{\lambda_{2}}\right)}^{2}+{\left(\frac{\lambda_{2}-\lambda_{3}}{\lambda_{3}}\right)}^{2}}$$ (3b)

The functional variables were acquired in five lobes (RUL, RLL, RML, LUL, and LLL) and whole lung (total). The spatial data analyzed included *D*_h_ and WT from TLC images, along with the Emph and fSAD both extracted from TLC images. To account for respiratory-induced deformation, image registration was performed to align FRC images within the TLC space. We further obtained CT-based inspiratory capacity (IC), as the volume difference between total lung capacity (TLC) and functional residual capacity (FRC) CT images acquired in the supine position, reflecting the tidal breathing feature, being similar to the slow vital capacity (SVC).

### EIT-based frequency and respiration variables

**FIGURE 5:**
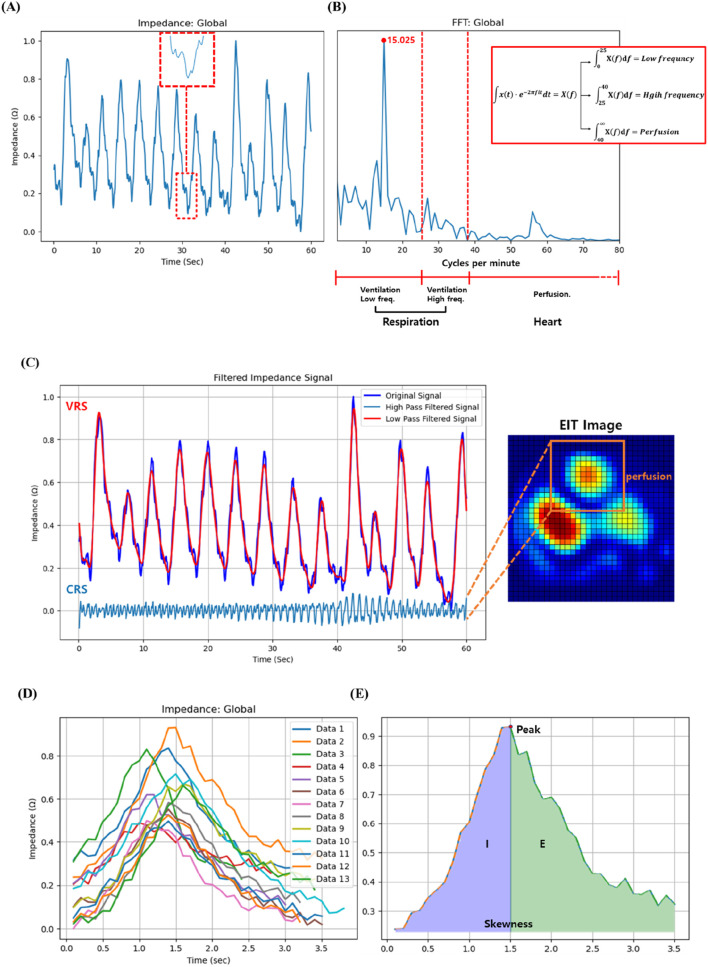
**(A)** Illustration of the high frequency found in the impedance change signal over time measured through EIT, **(B)** Schematic of the low, high frequency and perfusion after fast Fourier transform, **(C)** Perfusion signal separated from impedance change signal over time, **(D)** Multiple breathing cycles of a patient measured through EIT for 60 s, and **(E)** Inspiration (I):expiration (E) ratio and skewness of the patient.

The EIT system, configured with the standard adjacent injection pattern for the Dräger PulmoVista® 500, generated a time-series of impedance images at 10 frames per second. Each 32x32 cross-sectional image frame was reconstructed from a complete set of 208 voltage measurements acquired over approximately 1/10th of a second. From this image sequence, a global impedance waveform was derived. It is crucial to clarify that this waveform is not the signal from a single electrode pair. Instead, it is produced by integrating the impedance change values across the entire lung region of interest for each individual frame to yield a single summary statistic. This sequence of summary values, when plotted against time, forms the global impedance waveform, which represents the temporal change in overall lung aeration (Figure 5A). This waveform, reflecting tidal breathing, was then transformed into the frequency domain for analysis using the Fast Fourier Transform (FFT) method (Figure 5B) (Kerrouche et al., 2001). It is hypothesized that these frequency components have distinct physiological origins. The low-frequency signal (typically below 40 breaths per minute) originates from the large impedance changes caused by air filling and emptying from the lungs during the respiratory cycle, thus serving as a direct measure of regional ventilation. In contrast, the high-frequency signal, corresponding to the heart rate, originates from the smaller, pulsatile impedance changes caused by the flow of blood into the pulmonary vasculature with each heartbeat.

In this research, we adopted a modified approach to distinguish between perfusion and respiration by employing a filtering technique based on 2.5 times the peak respiration frequency as suggested by Caroline A. Grant et al. (Grant et al., 2011). Respiration components were defined as a frequency range of 2–40 breaths per minute, and thus frequencies exceeding 40 breaths per minute can be categorized as perfusion. More specifically, frequencies below 25 breaths per minute are in the low-frequency region, and respiration rates above 25 breaths per minute while resting may be a sign of underlying health. On the other hand, the frequency of 25–40 breaths per minute is in the high-frequency region, where there is abnormal signal absorption between normal and heart rates. The respiratory rates per minute of the study subjects were all less than 25, consistent with the findings from the Cleveland Clinic indicating that adults have a normal breathing rate of 12–25 breaths per minute at rest (no activity) (Clinica). Furthermore, the Cleveland Clinic suggests that heart rate typically ranges from 40 to 100 beats per minute, encompassing both sleep and exercise (Clinicb; Samaan, 2024). These criteria serve as pivotal elements for frequency distinction. In this study, the integration values within the three newly defined regions were designated as new variables for investigating their correlation with CT image variables in the study (Figure 5B).

Our choice of a 40 Cycles per minute cutoff to distinguish the lower-frequency ventilation signal from the higher-frequency cardiac signal is consistent with established practices in EIT analysis. For instance, prior studies have effectively used similar thresholds around 40 Cycles per minute to separate these two physiological signals, demonstrating that our selected cutoff is based on accepted methodologies in the field (Graf and Riedel, 2017). While we acknowledge that heart and respiratory rates can vary in diseased cohorts, a qualitative check was performed for each participant in this pilot study. We visually inspected the frequency power spectrum of the global EIT signal for each recording and confirmed a clear and unambiguous separation between the dominant, low-frequency respiratory peak and the secondary, high-frequency cardiac peak in all six cases. This provided confidence that our fixed threshold was adequate for robustly separating the two signal components in this specific dataset.

The respiration patterns measured by EIT were utilized to analyze the association with CT image variables. Each subject’s breathing was monitored for 60 s over multiple breath cycles, so that their skewness and integral values could be further computed (Figure 5E). The integral value was partitioned into left and right segments based on the peak, and the ratio of these areas was established as a variable, i.e., E:I ratio. In Figure 5E, left part (I) corresponds to inhalation, while the right part (E) corresponds to exhalation. While the value of I is relatively stable across subjects, there is considerable variability in E among patients. Therefore, the E:I ratio was calculated by dividing E by I. The correlation between CT image variables and the exhalation and inhalation (E:I) ratios was further investigated.

### Statistical analysis

Statistical analyses were conducted to explore the relationships among variables derived from EIT, CT, and PFTs variables. Spearman correlation coefficients (*ρ*) and their corresponding *P* values were computed to assess their associations. Furthermore, to account for the small sample size and aid in evaluating the strength and precision of the associations, 95% confidence intervals were also presented for all reported correlation coefficients. A significance level of *P* < 0.01 was taken. All data preprocessing and statistical analyses were performed using Python version 3.11.

## Results

### Breathing patterns of subjects with high FEV1/FVC vs. low FEV1/FVC

**FIGURE 6:**
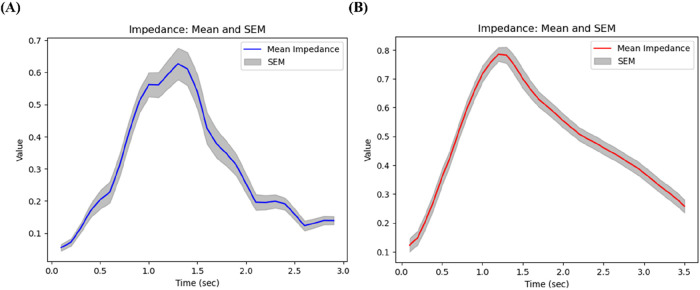
Breathing patterns of **(A)** a subject with relatively high FEV1/FVC (64%), and **(B)** a subject with low FEV1/FVC (32%).

The respiratory patterns showed distinct characteristics depending on the subject’s lung function status. First of all, we compared breathing patterns of two subjects with relatively high FEV1/FVC (64%) vs. low FEV1/FVC (32%). Figure 6A illustrates the respiratory pattern of patients with high PFTs, while Figure 6B represents the respiratory pattern of patients with low PFTs. Overall impedance values during inspiration seem to be similar between two subjects, but they were quite different during expiration. The subject with better lung function has sharp drop of impedance, but the subject with lower lung function has gradual decline of the slope. Thus, as lung function deteriorates, the expiratory phase tends to lengthen relative to the inspiratory phase. This prolonged expiratory phase in subjects with poor lung function suggests increased airway resistance, typical characteristics of asthma.

### Correlation between EIT and pulmonary function (PFTs and CT) results

**FIGURE 7:**
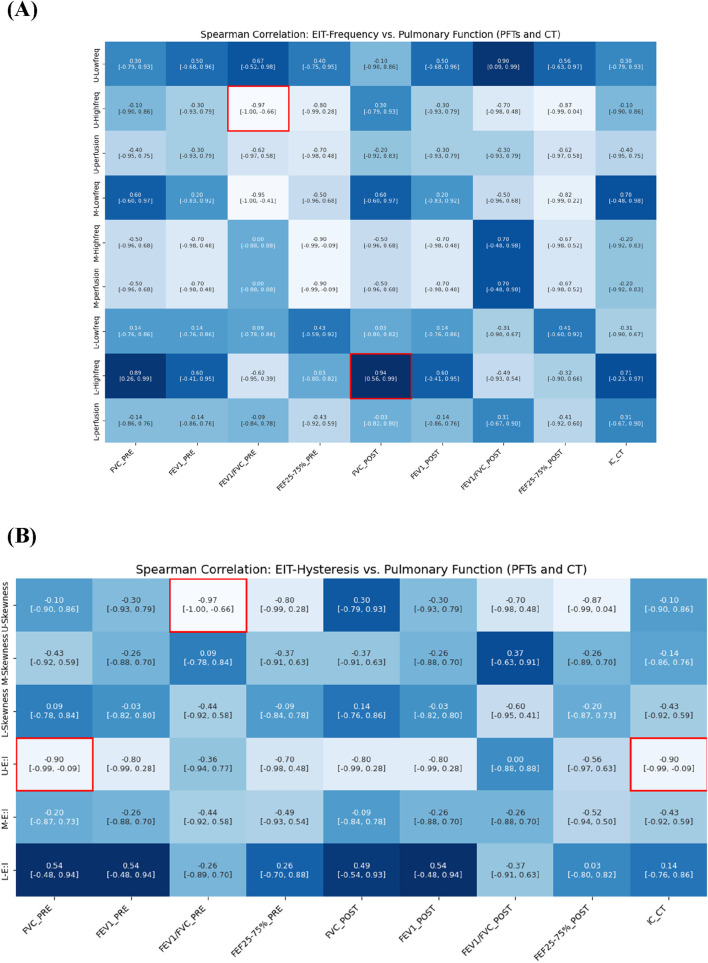
Spearman correlation of EIT-based **(A)** breathing frequency, **(B)** E:I ratio and skewness with pulmonary function metrics from PFTs and CT (e.g., IC).

Our analysis revealed significant relationships between electrical impedance measurements and conventional pulmonary function tests, particularly in the upper lung regions. In Figure 7, an increase in the high-frequency value of the upper lung (U-High, EIT) was associated with a decrease in the FEV1/FVC ratio (PFTs) at pre-bronchodilator. Similarly, an increase in the skewness of the upper lung (U-Skewness, EIT) was significantly correlated with a decrease in the FEV1/FVC ratio before bronchodilator.

We also analyzed CT-based IC, measured in the supine position, which reflects tidal breathing characteristics. The IC demonstrated a strong negative correlation with the E:I ratio in the upper lung (ρ = −0.90), a hysteresis-related variable. IC exhibited trends similar to FVC and showed direct relevance to the evaluation of lung function under tidal breathing conditions. Notably, IC effectively captured hysteresis parameters derived from EIT that reflect breathing patterns during tidal breathing.

### Correlation between EIT and CT-based structural variables

**FIGURE 8:**
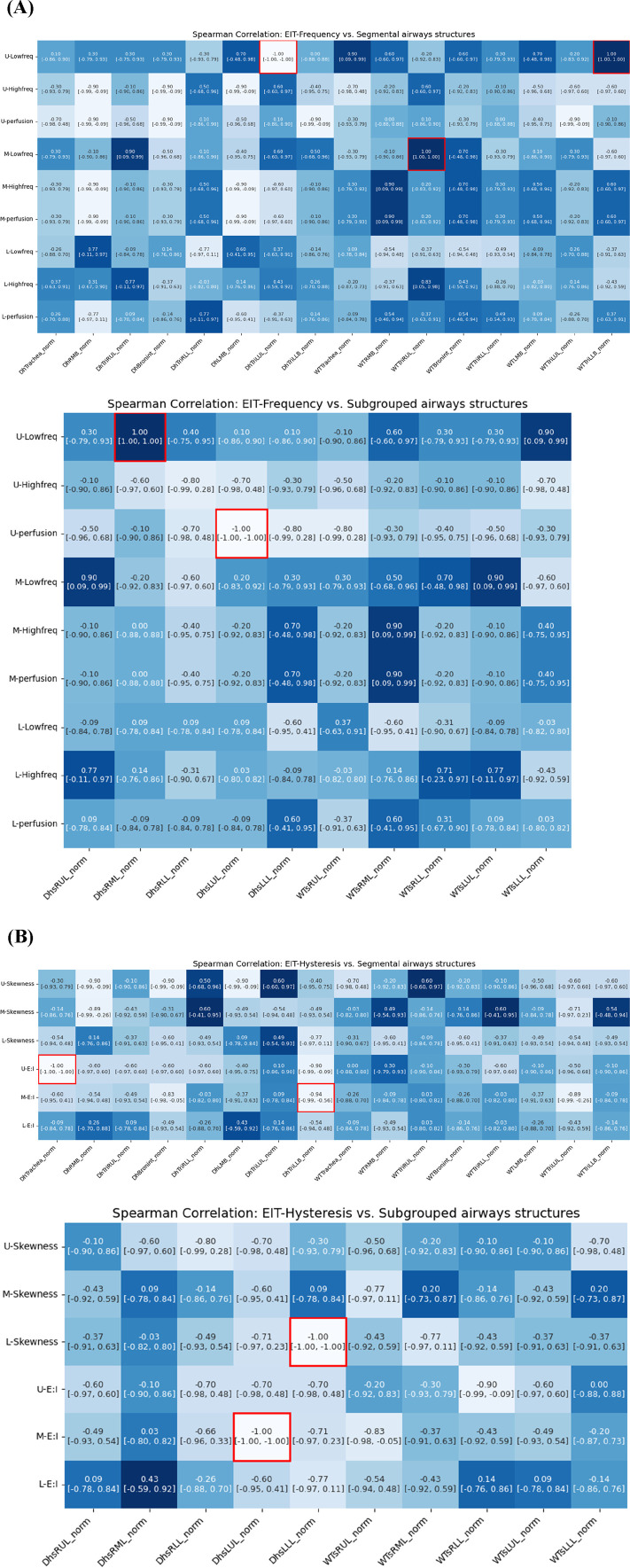
Spearman correlations of EIT-based **(A)** breathing frequency, **(B)** breathing hysteresis (E: I ratio and skewness) with CT segmental structural metrics.

The analysis demonstrated multiple significant correlations between airway dimensions measured by CT and ventilation distribution patterns measured by EIT. Figure 8 illustrates the correlation between CT-based structural parameters and EIT respiratory frequency parameters. In the main branches, an increase in the wall thickness (WT) of TriRUL and TriLLB (CT) was associated with an increase in the low-frequency value of the upper lung (U-Low, EIT). Similarly, in the sub-branches, an increase in the hydraulic diameter (*D*_h_) of sRML (CT) was associated with an increase in the low-frequency value of the upper lung (U-Low, EIT).

Among the respiratory hysteresis variables obtained through EIT, the exhalation-to-inhalation (E:I) ratio in the upper lung (U-E:I, EIT) showed a significant correlation with structural variables obtained through qCT. In the main branches, a smaller *D*_h_ in the trachea and TriLLB (CT) were associated with higher U-E:I values. In the sub-branches, the *D*_h_ of sLUL decreased with increasing E:I ratio in the middle lung lobe (M-E:I, EIT), while the *D*_h_ of sLLL decreased with increasing skewness in the lower lung lobe (L-skewness, EIT).

### Correlation between EIT and CT-based functional variables

**FIGURE 9:**
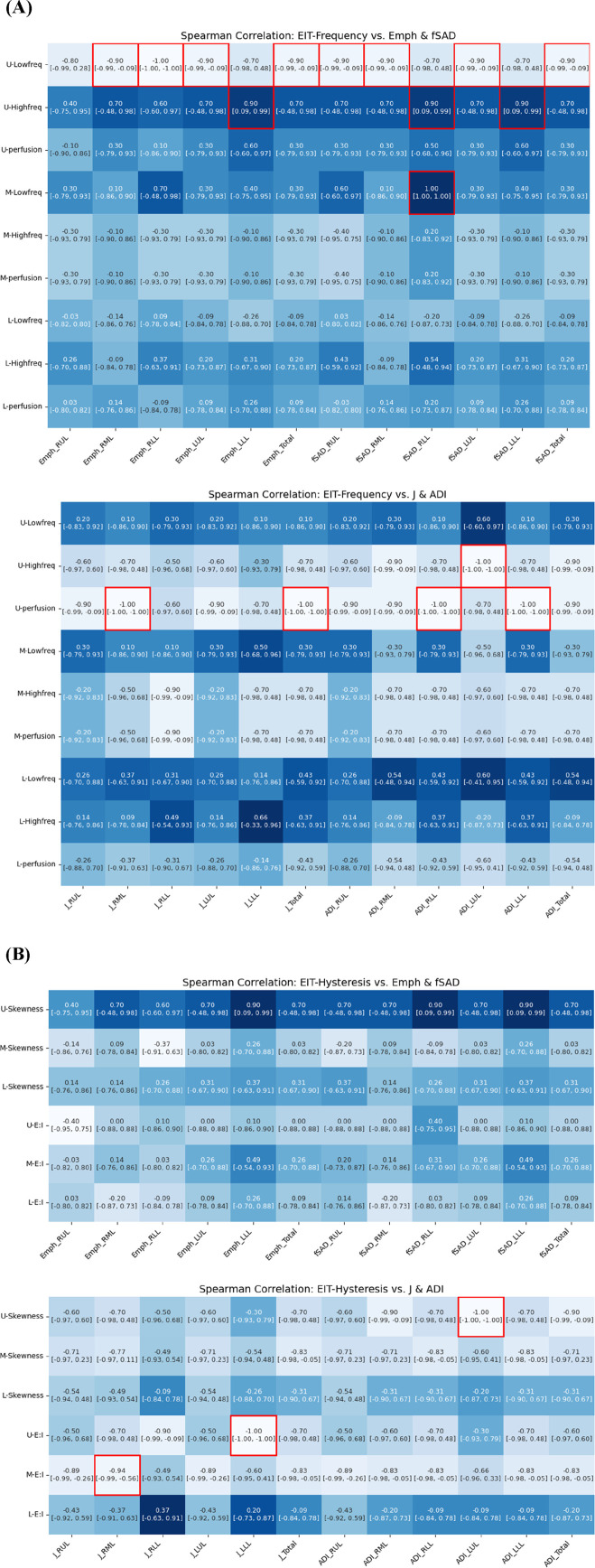
Spearman correlation of EIT-based **(A)** breathing frequency, **(B)** breathing hysteresis (E: I ratio and skewness) with CT parenchymal functional metrics.

Quantitative analysis revealed strong associations between regional ventilation patterns measured by EIT and lung tissue characteristics assessed by CT. Figure 9 illustrates significant associations between qCT-based functional variables and EIT-based indicators. An increase in EIT-based high-frequency values in the upper lung (U-High, EIT) showed a significant positive correlation with an increase in emphysema (Emph) in the left lower lobe (LLL). Additionally, U-High was strongly associated with an increase in functional small airway disease (fSAD) in the lower lung (ρ in the left lower lobe = 0.90, ρ in the right lower lobe = 0.90). Conversely, an increase in low-frequency values in the upper lung was correlated with a decrease in disease severity. These findings suggest that high-frequency components are significantly associated with structural and functional changes, as reflected by Emph and fSAD. Furthermore, an increase in U-High in the left upper lobe (LUL) was associated with a reduction in the anisotropic deformation index (ADI). Similarly, increased perfusion was correlated with decreases in both the Jacobian and ADI values.

Regarding respiratory hysteresis variables derived from EIT, an increase in U-Skewness was significantly associated with a decrease in ADI in the left upper lobe (LUL). Moreover, the Jacobian value in the LLL decreased with increasing U-E:I values, while the M-E:I value increased with a decrease in the Jacobian value in the right middle lobe (RML). These comprehensive correlations between EIT and CT parameters demonstrate that EIT can effectively capture both the structural and functional aspects of lung disease, particularly in detecting regional variations in ventilation and tissue characteristics.

## Discussion

For the purpose of comparing CT vs. EIT metrics, we prospectively collected a set of CT images including inspiration and expiration scans, and EIT data from six participants with asthma. Furthermore, PFTs were performed in pre- and post-bronchodilators. Using fast Fourier transform, the temporal metrics of impedances from EIT were transformed into frequency domains, characterized by low frequency, high frequency, and perfusion. We conducted Spearman’s correlations to explore the relationships among EIT, CT, and PFT metrics for better understanding pathophysiology from the temporal and spatial signals provided by different modalities.

Although tidal breathing and forced exhalation represent distinct respiratory patterns, both reflect the functional status of the same respiratory system. Fundamental physiological mechanisms influencing lung function—such as airway resistance, airway narrowing, lung elasticity, lung compliance, and the mechanical properties of the thoracic wall—are active in both breathing patterns. While correlations were observed between EIT-derived metrics obtained during tidal breathing and traditional PFT results, these findings must be interpreted with caution. Pulmonary function tests (PFTs) remain the gold standard for assessing global lung function. Our data do not suggest that EIT can replace spirometry. Rather, they indicate that integrating EIT with PFTs may offer a more comprehensive understanding of lung pathophysiology in asthma. EIT provides novel regional insights into the spatial and temporal heterogeneity of ventilation that are not captured by the global metrics of PFTs, thereby serving as a complementary—rather than substitutive—modality. Frequency domain analysis using Fourier transform enables the decomposition of impedance variation patterns over time, isolating contributions from specific frequency bands. These frequency components quantitatively reflect the patient’s overall pulmonary function, allowing for correlation analysis with pulmonary function test (PFT) results obtained from forced maneuvers. The correlation between tidal breathing and PFT results from forced maneuvers has been demonstrated in multiple studies (Frerichs et al., 2016; Hebbink et al., 2024; Qu et al., 2025; Cui et al., 2024; Vogt et al., 2016; Reinartz et al., 2019). Notably, both methods provide complementary information, particularly in assessing ventilation distribution. Furthermore, tidal breathing reflects a patient’s typical daily respiratory pattern, offering a different perspective on lung function compared to forced maneuvers. By analyzing the correlation between these two measurement techniques, it is possible to gain insights into the relationship between routine breathing conditions and maximal respiratory capacity. In this study, we compared CT-based IC with EIT metrics and observed similar correlation patterns. The IC, reflecting volume changes similar to SVC, demonstrated consistent associations with frequency-domain and hysteresis-related variables obtained from EIT, reinforcing the relevance of EIT-derived parameters in evaluating pulmonary function under spontaneous breathing conditions. Among the three anatomically segmented and measured regions, the upper part exhibited a distinct respiratory pattern, due to the fact that EIT measurements were taken during tidal breathing in the supine position. This study demonstrated that subjects with low-frequency dominance in EIT are likely to have better CT features, whereas subjects with high-frequency dominance are likely to have an alteration of CT features such as elevated Emph% and fSAD%.

Previous studies by Cristiano et al. and Binks et al. (Cristiano and Schwartzstein, 1997; Binks et al., 2001) suggest that stimulation of lung receptors by thoracic vibrations can potentially alter the perception of dyspnea. Additionally, Eckmann et al. (Eckmann and Gavriely, 1996) noted that these vibrations may enhance respiratory function by improving gas exchange within the central airways. In this study, the high-frequency variable in the upper lung showed a significant positive correlation with emphysema (Emph%) and functional small airway disease (fSAD%) in the lower lobes. Conversely, Jacobian and ADI—representing the magnitude and features of regional lung deformation—were negatively correlated with both the expiratory-to-inspiratory ratio and signal skewness. Although the precise physiological mechanism underlying these intra-thoracic associations remains unclear, one possible explanation involves cardiopulmonary interactions or the transmission of thoracic vibrations that influence regional ventilation dynamics. These high-frequency components may reflect compensatory or pathological processes related to dyspnea perception, gas exchange, and respiratory function. While causality cannot be established from the present data, our findings offer a novel perspective on inter-lobar relationships in obstructive lung disease and underscore the need for further research to explore these complex interactions. The EIT-derived perfusion signal, which is influenced by the cardiac rate, was found to correlate with Jacobian and ADI values—metrics that reflect the degree of lung deformation. It is known that in asthma, complex processes involving hypoxia, sympathetic nervous system activation, and bronchoconstriction contribute to ventilation-perfusion mismatch. Our observed correlation between the EIT perfusion signal and deformation metrics could potentially be a reflection of these underlying processes. However, we must state clearly that this is a hypothesis. Our study was not designed to establish a mechanistic link, and further investigation is required to understand the precise relationship between cardiac-related EIT signals and regional lung mechanics in asthma (Bhandary, 2015; Gayen et al., 2024; Tanabe et al., 2024; Hopkins, 2020).

Furthermore, exhalation-to-inhalation ratio (E:I), a breathing hysteresis variable derived from EIT, consistently correlated with the structural variable *D*_h_ from CT across the both main and sub-branches. It was found that the larger the E:I variable obtained through EIT, the smaller hydraulic diameter. For respiratory patterns in normal subjects, the ratio of E:I is relatively shorter. The normal E:I for spontaneously breathing patients is typically about 3:1 to 5:1, i.e., the expiratory time ratio is 3–5 times longer than the intake time ratio (F. L. MD). The correlation between CT and EIT was reconfirmed by the PFT results. The FEV1/FVC results are consistent with functional parameters observed in high-frequency vibrations of the upper lobes. Furthermore, an elevated U-skewness variable indicates impaired lung function, as evidenced by the PFT results. This finding consistently correlates with a reduced *D*_h_, increased fSAD, and decreased ADI values. These results suggest that the skewness variables derived from EIT may reflect both the structural and functional characteristics observed in CT imaging and Pulmonary function tests.

This study has several limitations. The most significant is the small sample size of six participants, which inherently limits the statistical power and the generalizability of our findings. This study was conceived as a pilot or hypothesis-generating study, with the primary goal of assessing the feasibility of integrating two complex imaging modalities—EIT and qCT—and generating preliminary data for future, larger-scale validation studies. The small cohort was a result of the practical constraints of a 1-year institutional research project. To address the limitations of the sample size and to aid in the interpretation of the strength and precision of the associations, we have reported 95% confidence intervals for all correlation coefficients. Consequently, all interpretations and conclusions are presented cautiously within the exploratory nature of this research. We emphasize that these preliminary results require rigorous verification through future studies with sufficient statistical power.

A key limitation of our study is the lack of correction for multiple comparisons. Given the large number of correlations performed, this increases the risk of Type I errors. However, due to the exploratory nature and very small sample size of this pilot study (N = 6), we prioritized the exploration of potential trends over the strict control of false positives, which could have masked important signals due to low statistical power. Therefore, the findings presented here should be interpreted with caution as preliminary results that require validation in future, larger-scale studies.

A second limitation pertains to the spatial correspondence between the three vertical EIT electrode planes and the actual lung lobes. We acknowledge that assuming a direct, one-to-one mapping is an oversimplification, dictated by the inherently low spatial resolution of EIT as a functional imaging modality. This simplification introduces a potential for spatial misregistration, where signals from one lung region could be attributed to an adjacent measurement plane. As stated in the Methods, our primary goal was to assess broad functional gradients along the vertical axis rather than to achieve precise lobar segmentation. Nevertheless, we have added this assumption as a key limitation, as it may compromise the spatial accuracy of signal attribution.

A third limitation pertains to the methodological interpretation of the EIT frequency components. We acknowledge the inherent complexities and potential for spectral overlap in this technique. Crucially, the perfusion-related signal is an indirect surrogate and does not represent a direct measurement of absolute blood flow. The validity of this signal as a quantitative perfusion metric is an area of ongoing research, as it can be influenced by factors other than blood flow, such as vascular tone, airway pressure, and the mechanical motion of the heart itself. However, while recognizing this ambiguity, we maintain that investigating the correlation between these frequency-separated signals and structural changes seen on CT is a valid and valuable exploratory approach. It allows us to generate novel hypotheses about how ventilation and perfusion patterns relate to the underlying pathophysiology in asthma.

Additionally, this study utilized fixed frequency thresholds to separate signal components without a formal sensitivity analysis. While our qualitative visual inspection confirmed clear signal separation in our cohort, we recognize that these fixed thresholds may not be universally optimal for all patients, especially in diverse diseased populations with significant variability in heart and respiratory rates. This represents a methodological limitation, and future studies could be improved by implementing patient-specific adaptive filtering techniques to more robustly account for such inter-individual variability.

These findings offer a perspective on how EIT-derived temporal signals can provide complementary insights into pulmonary dynamics, which are not captured by static quantitative CT variables. By highlighting the relationship between regional EIT metrics and both structural (CT) and global functional (PFT) assessments, this work suggests that EIT may be a useful tool alongside established methods, potentially paving the way for a deeper, more integrated understanding of the respiratory system in asthma.

## Data Availability

The original contributions presented in the study are included in the article/supplementary material, further inquiries can be directed to the corresponding authors.

## Glossary

**qCT**: quantitative Computed Tomography
**EIT**: Electrical Impedance Tomography
**PFTs**: Pulmonary Function Tests
**Emph**: Emphysema
**fSAD**: functional Small Airway Disease
**ADI**: Anisotropic Deformation Index
**WT**: Wall Thickness
**D**_**h**_: hydraulic Diameter
**D**_**out**_: outer Diameter
**D**_**ave**_: average Diameter
**LA**: Luminal Area
P_e_: Perimeter of the luminal area
**FEV1**: Forced Expiratory Volume in one second
**FVC**: Forced Vital Capacity
**FEF25%-75%**: Forced Expiratory Flow between 25% and 75% of vital capacity
**IC**: Inspiratory Capacity
**SVC**: Slow Vital Capacity
**U-High**: Upper lung High frequency
**U-Low**: Upper lung Low frequency
**U-perfusion**: Upper lung perfusion rate
**M-High**: Middle lung High frequency
**M-Low**: Middle lung Low frequency
**M-perfusion**: Middle lung perfusion rate
**L-High**: Lower lung High frequency
**L-Low**: Lower lung Low frequency
**L-perfusion**: Lower lung perfusion rate
**U-E:I**: Upper lung ratio of Expiratory to Inspiratory
**M-E:I**: Middle lung ratio of Expiratory to Inspiratory
**L-E:I**: Lower lung ratio of Expiratory to Inspiratory
**U-skew**: Upper lung skewness
**M-skew**: Middle lung skewness
**L-skew**: Lower lung skewness
**FFT**: Fast Fourier Transform
**RUL**: Right Upper Lobe
**RML**: Right Middle Lobe
**RLL**: Right Lower Lobe
**LUL**: Left Upper Lobe
**LLL**: Left Lower Lobe
**BronInt**: Bronchus Intermedius
**RMB**: Right Main Bronchus
**LMB**: Left Main Bronchus
**TriRUL**: Trifurcation of Right Upper Lobe
**TriLUL**: Trifurcation of Left Upper Lobe
**sRUL**: subgrouped Right Upper Lobe including branches of RB1 to RB3
**sRML**: subgrouped Right Middle Lobe including branches of RB4 and RB5
**sRLL**: subgrouped Right Lower Lobe including branches of RB6 to RB10
**sLUL**: subgrouped Left Upper Lobe including branches of LB1 to LB5
**sLLL**: subgrouped Left Lower Lobe including branches of LB6 to LB10
**BMI**: Body Mass Index
**LAMA**: Long-Acting Muscarinic Antagonists
**LABA**: Long-Acting β2-adrenergic Agonists
**SABA**: Short-Acting β2-adnergic Agonists
**ICS**: Inhaled Corticosteroids
**OCS**: Oral Corticosteroids
**WBC**: White Blood Cell
**IgE**: Immunoglobulin E
**FeNO**: Fraction of exhaled Nitric Oxide
